# The power of mouse models in the diagnostic odyssey of patients with rare congenital anomalies

**DOI:** 10.1007/s00335-025-10114-2

**Published:** 2025-03-18

**Authors:** Stephen R. F. Twigg, Nicholas D. E. Greene, Deborah J. Henderson, Pleasantine Mill, Karen J. Liu

**Affiliations:** 1 MRC National Mouse Genetics Network, Congenital Anomalies Cluster, Mary Lyon Centre at MRC Harwell, UK; 2 MRC Weatherall Institute of Molecular Medicine, John Radcliffe Hospital, University of Oxford, Oxford, UK; 4 Biosciences Institute, Centre for Life, Newcastle University, Newcastle upon Tyne, UK; 5 MRC Human Genetics Unit, MRC Institute of Genetics & Cancer, University of Edinburgh, Edinburgh, UK; 6 Centre for Craniofacial and Regenerative Biology, King’s College London, London, UK

**Keywords:** Congenital anomalies, Variant of uncertain significance, Mouse models, Genotype-to-phenotype correlation, Molecular diagnosis, Diagnostic odyssey

## Abstract

Congenital anomalies are structural or functional abnormalities present at birth, which can be caused by genetic or environmental influences. The availability of genome sequencing has significantly increased our understanding of congenital anomalies, but linking variant identification to functional relevance and definitive diagnosis remains challenging. Many genes have unknown or poorly understood functions, and with a lack of clear genotype-to-phenotype correlations, it can be difficult to move from variant discovery to diagnosis. Thus, for most congenital anomalies, there still exists a “diagnostic odyssey” which presents a significant burden to patients, families and society. Animal models are essential in the gene discovery process because they allow researchers to validate candidate gene function and disease progression within intact organisms. However, use of advanced model systems continues to be limited due to the complexity of efficiently generating clinically relevant animals. Here we focus on the use of precisely engineered mice in variant-to-function studies for resolving molecular diagnoses and creating powerful preclinical models for congenital anomalies, covering advances in genomics, genome editing and phenotyping approaches as well as the necessity for future initiatives aligning animal modelling to deep patient multimodal datasets.

## Congenital anomalies are a significant global challenge

Approximately 2–4% of babies are born with one or more severe anatomical malformations (Bacino, 2023; https://digital.nhs.uk/data-and-information/publications/statistical/ncardrs-congenital-anomaly-statistics-annual-data) with a significant proportion dying within 28 days of birth (https://www.who.int/news-room/fact-sheets/detail/birth-defects). With recent advances in sequencing technology, identification of potentially causative genetic alterations is increasing rapidly. However, it remains a major bottleneck to establish mechanistic links between genetic changes and the observed clinical presentations. Pinpointing which of the identified inherited or spontaneous changes in a patient’s DNA is responsible for disrupting normal prenatal development can be challenging - this includes variants in genes not previously associated with disease, variants in known disease genes but with novel clinical features, and more commonly variants of uncertain significance (VUS), including in non-coding regions, where pathogenicity is difficult to predict or model.

Understanding how these potentially pathogenic variants impact normal development is also difficult. Many of the genes implicated in congenital anomalies play recurring roles in different tissues and across developmental stages *in utero* or in early life. As such, these genes are difficult to study in humans, even in ‘disease-in-a-dish’ human stem cell or organoid models. Precisely engineered animal models of patient variants are needed to replicate complex interactions disrupted during morphogenesis, often across multiple organ systems. Moreover, disease modelling in model organisms allows us to better understand the genotype-phenotype variability observed in our patient cohorts, by allowing us to modulate genetic background/modifiers and environment to tackle pleiotropy and penetrance. The grand challenge for the functional genomics of congenital anomalies is the coordination across disciplines, scales and systems to effectively deliver harmonized analyses of multiple body systems. This necessitates close alignment between human rare disease genetics and advanced genome engineering in model organisms coupled to deep clinical and model phenotyping in order to feedback actionable information to clinicians and patients.

### The diagnostic odyssey in rare congenital diseases

Many congenital anomalies fall into the category of rare diseases. These conditions can be complex, with multi-system or syndromic phenotypes which complicate the diagnostic process. Unbiased genetic sequencing approaches, such as exome and genome sequencing, can be a powerful way to rapidly screen for clinically relevant variants in a diagnostic capacity, capturing both known and new genetic disorders in a significant proportion of patients. Despite these improvements in genetic technologies, the path to diagnosis - commonly referred to as the “diagnostic odyssey” - remains arduous for many patients and their families. This is due in part to difficulties in validating the functional relevance of identified gene variants, which greatly limits diagnostic accuracy. The American College of Medical Genetics and Genomics (ACMG) and the Association for Molecular Pathology (AMP) have established criteria for classification of genetic variants. Insufficient evidence for identified variants results in them failing to be assigned as pathogenic or likely pathogenic using ACMG/AMP criteria to confirm or be consistent with a molecular diagnosis (Richards et al. 2015). Diagnostic yields differ depending on whether an exome or genome sequencing approach is taken, with an uplift of 7–25% in diagnostic rate reported for genome sequencing (Alfares et al. 2018; Lionel et al. 2018; Helman et al. 2020; Ewans et al. 2022; Wojcik et al. 2024), and on the range of conditions being tested. The Deciphering Developmental Disorders (DDD) study has a diagnostic rate of ~ 35% from exome analysis of developmental disorders (https://www.nihr.ac.uk/story/ddd-study-deciphering-developmental-disorders-study), while in the UK 100,000 Genomes Project a genetic diagnosis has been made in ~ 25% rare disease probands after genome sequencing (Smedley et al. 2021). In congenital disorders diagnostic rates vary from around 1–10% in conditions with etiologically complex disorders with environmental as well as genetic risk factors such as spina bifida (Vong et al. 2024) and orofacial clefting (Diaz Perez et al. 2023), up to 30–40% in conditions such as craniosynostosis (Hyder et al. 2021) and congenital heart disease (Morrish et al. 20221), with the highest yields for syndromic presentations.

Accelerating molecular diagnosis can lead to earlier access to treatment to improve outcomes for the fetus and mother. Many congenital structural anomalies can be observed *in utero*, and in these cases where a genetic cause is suspected, non-invasive prenatal genome sequencing can be incorporated into prenatal diagnosis and care plans (for example the R21 pathway for rapid exome sequencing available in the UK NHS; Vora and Norton 2023). The diagnostic utility of targeted or exome sequencing for prenatal diagnosis of unselected fetal structural abnormalities in two large studies reported diagnostic rates of 8.5–10%, with the rates increasing with the number and severity of anomalies detected (Petrovski et al. 2019; Lord et al. 2019). Diagnostic rates differed depending on selected ultrasound anomaly features from 41 to 53% diagnostic yield for isolated skeletal anomalies to 2% for isolated increased nuchal translucency (Xue et al. 2024; Mellis et al. 2022). Although simply including routine sequencing of pregnancies with detected structural anomalies will greatly improve diagnostic efficiency, diagnostic accuracy is impossible when functional evidence for identified variants is limited or absent. Indeed, most identified variants are categorised as VUS and remain uncharacterized. More sequencing alone will not help overcome this bottleneck. Furthermore, the absence of suitable preclinical models poses significant challenges for deciphering disease mechanisms and assessing therapeutic avenues.

### Critical challenges in identifying the underlying causes of congenital anomalies

Interpreting the clinical significance of identified genetic variants necessitates interdisciplinary collaboration involving clinicians, geneticists, bioinformaticians, and other specialists. A collective approach ensures that phenotypic data, variant classification, and clinical judgment are integrated effectively. Many of the candidate genes lack comprehensive annotations, making it difficult to predict their roles. Initiatives like the NIH-based ClinGen and ClinVar focus on establishing standards for variant interpretation, resolving conflicting data, and fostering collaboration across laboratories and clinicians. For congenital anomalies, DECIPHER (DatabasE of genomiC varIation and Phenotype in Humans using Ensembl Resources; https://decipher.sanger.ac.uk/) is particularly useful, as it combines plausibly causative variants with well-phenotyped patients with rare diseases (Foreman et al. 2022). These projects emphasize the importance of sharing data and integrating expertise to clarify the functional role of genetic variants in human development and disease.

Key challenges to disease attribution in variant calling include pleiotropy, where a single gene can lead to diverse phenotypes when mutated, incomplete penetrance, and genetic heterogeneity, where a single feature or phenotype can arise from mutations in multiple genes. Gene families can also exhibit functional redundancy, where multiple genes perform similar functions, potentially masking the effects of specific loss-of-function type variants, although this may vary in a tissue or temporal manner. Gene expression and function are also influenced by epigenetic modifications and environmental factors which may affect penetrance of a particular feature. While we know non-coding variants may affect regulatory elements such as enhancers to affect gene expression, often in cell-type specific manners, these types of variants will generally be missed by exome-based approaches.

To further complicate matters, many conditions that are thought to be common are in fact not homogeneous. For example, anomalies such as craniosynostosis, cardiac and neural tube defects are relatively common in the general population, but, individual cases within each type of anomaly may be caused by different, rare, single-gene mutations. Thus, from a genetic perspective, each of these conditions could be considered groups of rare diseases. As a consequence, while these are managed collectively from a public health standpoint, each patient would benefit from personalised treatments and genetic counselling (Khokha et al. 2017).

Beyond genetics, accurate and consistent phenotyping of patients with congenital anomalies can be a major challenge. Deep phenotyping of patients, not just models, is key to understanding genotype to phenotype relationships, and inaccurate or insufficient clinical phenotyping can hinder recognition of differential diagnoses and the link to genotype. In many cases, documentation of phenotypic features may be incomplete or mild forms may overlap with ‘normal’ variation. Anomalies can present with varying degrees of severity or varying combinations of tissue involvement, leading to inconsistent disease classifications. Assessment of evidence from animal models is also complicated, as phenotypes between species are often incongruent (Henderson et al. 2024). Altogether, these complexities lead to a lack of clear genotype-phenotype correlations, particularly for congenital anomalies, which affect multi-organ functions that are impossible to replicate in a tissue culture dish.

### The importance of mouse models in rare disease research

Understanding the requirement for a given gene in human development and disease has been accelerated through mouse loss-of-function knockout (KO) studies providing invaluable insights into how our genome functions. Resources such as the International Mouse Phenotyping Consortium (IMPC) and programmes such as the Monarch Initiative (Putman et al. 2024; https://monarchinitiative.org/) have been instrumental in cataloguing mouse KO data and integrating it with human disease phenotypes. Mouse models can also serve as essential tools for studying the functional consequences of gene variants unbiasedly as powerfully demonstrated by forward genetic ENU mouse mutagenic screens, which can link specific point mutations to functional genomics for a wide range of multisystem phenotypes (Justice et al. 1999; Hrabé de Angelis et al. 2000; Herron et al. 2002). These complementary approaches have generated valuable allelic series for understanding functional genomics of mammalian development and disease, particularly for recessive conditions.

Rare congenital anomaly and disease genetics in human patients offer a similarly powerful opportunity to understand how our genes function in complex developmental processes. By recreating precise patient variants in mice using genome editing technologies, researchers can observe resulting phenotypes and compare them to human disease manifestations enabling the validation of variants linked to specific rare diseases. This is particularly useful for unpicking how different missense variants in the same gene can lead to grossly different features in patients with dominant conditions (Dodd et al. 2024). Mice carrying patient variants that accurately replicate disease phenotypes remain essential for pre-clinical therapeutic modelling, offering windows into disease progression and opportunities for designing therapeutic interventions for halting or reversing disease phenotypes. Robust mouse models of human rare disease remain essential pieces in the pathway to translation through gene-agnostic pharmacological interventions or increasingly gene-based therapeutics.

Table 1 summarises common publicly available resources for mouse developmental biology, and their relevance to human disease. By integrating genetic data from large sequencing efforts with patient phenotypes and linking these to resources such as the IMPC and Monarch Initiative datasets, researchers can leverage a wealth of information to better understand developmental models of human disease and accelerate the discovery of therapeutic targets.

Despite the availability and usefulness of mouse (Table 1) and other model system data (Table 2) connecting clinical observations with animal model data requires interdisciplinary collaboration and data integration (examples shown in Table 3**)**. In the UK, current efforts such as the MRC National Mouse Genetics Network (NMGN) Congenital Anomalies Cluster (https://nmgn.mrc.ukri.org/clusters/congenital-anomalies/) are establishing an integrated communication strategy focused on clinical-research collaborations equipped to curate data on human congenital anomalies. Linking the identification of novel VUS directly to precisely engineered mouse models that better the mimic human variant, offers potential to accelerate diagnostics and translation of preclinical models towards interventions for patients.

### Model system choice

In the age of functional genomics, moving from identification of variants to how they influence biological processes, the choice of model systems should be determined by the questions being asked. Gene-specific deep mutation scanning (DMS) allows endogenous assessment of all possible variants in key clinically relevant genes, such as tumour suppressors *BRCA1, BRCA2* or *p53* using genome editing (Findlay et al. 2018; Huang et al. 2023; Funk et al. 2025). But multiplexed assays of variant effect (MAVEs) can also be used to quantify the molecular phenotypes or functional impact of many thousands of genomic variants in a gene of interest or human disease protein domains of interest in a single overexpression experiment. They can be performed in either model organisms like yeast or in cell lines, where the cells are then screened for a phenotype of interest, often growth or reporter expression (Beltran et al. 2025; Fayer et al. 2021). While useful to unpick which variants affect protein stability and levels towards more accurate clinical variant classification, these assays cannot reveal why different cell types are affected or different developmental stages are vulnerable to the effect of a given variant. This is often key to understanding congenital anomalies.

Whilst the mouse is one of the most accessible and economical models at scale to allow systems-based understanding of the consequences of human variants in congenital anomalies, there are limitations. The use of inbred strains in mouse models significantly limits genetic diversity. Unlike outbred populations, inbred mice are nearly genetically identical, which can reduce variability in experimental outcomes but does not accurately represent the genetic diversity of human populations. Thus, the use of congenic strains reduces the applicability of findings to the broader human population, where genetic differences influence disease susceptibility and treatment responses. However, further insight into human disease manifestation can be gained by analysing the same mutation across backgrounds. Examples include the susceptibility of different strains to neural tube closure defects or hydrocephaly that influences phenotypic spectrum or severity (Fleming and Copp 2000; McKenzie et al. 2018), or the different phenotypes present in mice with deletion of *Tcof1* (Dixon and Dixon 2004) or a specific point mutation in *Fgfr3* (Twigg et al. 2009). The use of genetically diverse mouse populations (e.g., Collaborative Cross mice) can better mimic the heterogeneity observed in human populations, leading to findings that are more translatable to clinical outcomes. Resources such as the Mouse Phenome Database, hosted by Jackson labs, provide data on phenotypic variation in health and disease (Bogue et al. 2023).

While highly similar, the genomes of humans and mice do differ. Whilst on average the coding region of human and mouse genomes are 85% conserved, the non-coding genome is much less similar (< 50%) with genomic differences (such as gene expansions or losses, and rearrangements) as well as regulatory differences (such enhancers or splicing) leading to differences in gene expression levels and domains. As such modelling precise patient variants in mice may be more challenging if regulatory elements or even genes are not conserved. It has been estimated that around 20–30% protein-coding genes are either in one-to-many or many-to-many orthologous relationships in humans and mice, where species-specific expansions or reductions of genes have occurred, or contain species-specific open reading frames (Breschi et al. 2017). In some models, it is possible to ‘humanise’ the mouse by replacement of coding sequences, or the full human gene to preserve splicing, expression and biochemistry (Devoy et al. 2021). However, in order to understand how variants in other genes cause human disease phenotypes, additional steps such as expression studies in human embryos (Human Developmental Biology Resource, http://hdbr.org/; Table 3) or use of alternate human organoid/assembloid models may be necessary (Onesto et al. 2024). While human and mouse gene expression is often highly conserved at the level of gene regulatory networks, this is not true for conservation of regulatory sequences including promoters and enhancers or individual transcription factor binding sites (Breschi et al. 2017). Genome engineering in mouse and model organisms is still useful with alternate screening approaches for assessing how disease variants in human enhancers affect gene expression levels and domains in vivo for congenital anomalies (Hollingsworth et al. 2025; Bhatia et al. 2021).

The power of artificial intelligence and machine learning for mining disparate multi-scale datasets encompassing clinical to model organism phenotypes is rapidly evolving. Use of these approaches will improve efficient curation of genotype-phenotype relationships. Cross-species phenotyping, notably the automated ontology-based approaches currently being developed by the Monarch Initiative, an international open science consortium, look to connect key data sets across disciplines to accelerate rare disease diagnosis (Putman et al. 2024). Similarly, by systematically matching patient symptoms to analogous traits and genetic modules observed in other species, we can identify “phenologs” that can improve our disease gene prioritisation. Reciprocally, deep phenotyping data from mice carrying clinical variants may uncover additional systems affected in patients. In the future, improvements in computational predictions will support better integration of the diverse multiscale biological data necessary for efficient functional genomics.

### Beyond diagnosis: the challenge of treating rare and common congenital diseases

Even after a diagnosis is established, the rarity and complexity of these conditions often impede therapeutic development. Limited patient populations for rare disease, genetic variability among common congenital anomalies, and insufficient understanding of disease mechanisms often hinder progress. Additionally, the lack of financial incentives for pharmaceutical companies to invest in therapies for small patient groups exacerbates these challenges.

Preclinical therapeutic platforms rely heavily on the availability of relevant disease models and genetically tractable mouse models play a pivotal role in this context. Therefore, the development of more accurate and disease-specific models is urgently needed to provide a robust foundation for testing therapeutic strategies. In an age of genomic medicine, mice that precisely model patient variants prove extremely powerful for preclinical studies in developing genetic therapies to target the genetic defect directly using nucleic acid therapies (NATs) (Wang et al. 2020; Alter et al. 2006), gene therapies (LNP-mRNA or viral) or genome editing approaches (Bjursell et al. 2018; Maino et al. 2021). Alternatively, targeted approaches may focus on downstream pathways such as in utero administration of DKK inhibitors in preclinical studies using humanized mouse models of *FZD2* of Robinow syndrome, which showed ability to normalize skeletal growth (Liegel et al. 2023). However, these strategies require precise targeting, thorough safety evaluations, and robust delivery mechanisms. Disease-specific models play an essential role in these processes by enabling preclinical testing and refinement. As therapeutic options advance, ensuring equitable access to these cutting-edge treatments remains a priority.

In summary, while the diagnosis of rare congenital diseases has improved with advances in genomics, with leaps in the speed of genetic diagnosis, in informing patient management and mitigating future risks in families, there remains sizeable work to be done to understand how our genomes are wired during normal development. This is key to not just understanding disease mechanisms, but also in moving forward towards treating these conditions through innovative and collaborative approaches such as that proposed by the MRC NMGN Congenital Anomalies Cluster. Understanding the genetics of rare congenital diseases in diverse populations is also needed, necessitating global efforts to bridge gaps in fetal and maternal healthcare delivery. A major focus must be on improving the efficiency of gene discovery and functional genomics, while developing accurate disease models and harnessing emerging technologies are key steps toward addressing the unmet needs of this vulnerable population.

## Figures and Tables

**Table 1:** Resources for mouse developmental biology and their relevance to human disease

Resource	Description	Focus	Relevance to Human Disease	Website/Link
Mouse Genome Informatics (MGI)	Comprehensive resource for genetic, genomic, and phenotypic data on laboratory mice.	Provides data on gene expression, mutations, and phenotypes in mice.	Links mouse models to human disease phenotypes using orthologs.	MGI
International Mouse Phenotyping Consortium (IMPC)	Aims to systematically phenotype every gene in the mouse genome.	Mouse knockouts and phenotypes	Links mouse phenotypes to human genetic diseases.	IMPC
EMAP/EMAGE	Database for 3D anatomy and gene expression in mouse embryos.	Mouse embryonic development stages and gene expression maps.	Allows comparison of developmental gene expression between mouse and human.	EMAP/EMAGE
Developmental Genomics Atlas (DGA)	Data on gene expression during various developmental stages in mice.	Provides temporal and tissue-specific expression data in mouse embryos.	Useful for studying genetic basis of congenital diseases in humans.	DGA
Allen Brain Atlas	Spatial and temporal gene expression data for mouse and human brains.	Mouse brain developmental tsages and gene expression profiles.	Comparative insights into neurodevelopmental disorders in humans.	Allen Brain-Atlas
GUDMAP	Resource for genitourinary development and disease, providing gene expression data.	Developmental data on mouse urogenital systems.	Links developmental pathways to congenital and acquired human diseases.	GUDMAP
The Jackson Laboratory (JAX)	Repository of genetically engineered and inbred mouse strains for human disease research.	To discover the genetic basis of human disease using mouse models	Mouse Models for Human Disease Database cataloging developmental and genetic conditions with phenotypic descriptions.	www.jax.org
Eurexpress	Database of gene expression in the developing mouse embryo.	Maps spatial and temporal gene expression patterns to support studies on embryogenesis and developmental disorders.	Links transcriptomic atlas data for mouse embryo	eurexpress.org

**Table 2:** Resources for non-mouse animal models

Resource Name	Description	Model System	Relevance to Human Disease	Website/Link
Alliance of Genome Resources	Consortium of genome data and resources for multiple model organisms	Multiple	Model organism and human comparative genomics	Alliance of Genome Resources
FlyBase	Genomic and biological data for *Drosophila melanogaster.*	Fruit fly (*Drosophila melanogaster*)	Studies fundamental biological processes and their relation to diseases.	FlyBase
Rat Genome Database (RGD)	Genetic, genomic, and phenotypic data on rats.	Rat	Models cardiovascular, neurological, and metabolic diseases.	RGD
WormBase	Genomic and phenotypic data for *Caenorhabditis elegans.*	Nematode (*C. elegans*)	Insights into genetic regulation, aging, and neurodegenerative diseases.	WormBase
Xenbase	Database for *Xenopus* genomic, transcriptomic, and phenotypic data.	Frog (*Xenopus laevis* and *X. tropicalis*)	Studies vertebrate development, organogenesis, and disease mechanisms.	Xenbase
ZFIN (Zebrafish Information Network)	Comprehensive resource for zebrafish development and genetics.	Zebrafish (*Danio rerio*)	Studies vertebrate development and genetic contributions to diseases.	ZFIN
Yeast Genome Database (SGD)	Data for *Saccharomyces cerevisiae* (budding yeast).	Budding yeast (*S. cerevisiae*)	Focuses on basic cell processes relevant to cancer and metabolic diseases.	SGD

**Table 3:** Resources for comparative human disease modelling: resources dedicated to comparing different species to model human diseases and disorders. These platforms integrate multi-species data to identify conserved genetic, phenotypic, and pathological features, enhancing our understanding of human biology and disease mechanisms

Resource	Description	Model Species Inclusion	Relevance to Human Disease	Website/Link
OMIM (Online Mendelian Inheritance in Man)	Catalog of human genes and genetic disorders.	Not mouse-specific but used alongside mouse models to study diseases.	Links mouse models to human genetic disorders through orthologous genes.	OMIM
Ensembl Genomes	Comparative genomics platform for various species.	Various (plants, bacteria, animals)	Links genomic information across species to human biology and diseases.	EnsemblGenomes
Human Developmental Biology Resource	Tissue bank and atlas of human development	Human	Enables analysis of anatomical structures with mapped gene expression data, and access to tissues	HDBR
Human Protein Atlas	Protein expression data in humans and various model organisms.	Human, mouse, pig	Provides insights into protein function and its role in human disease.	Human-Protein Atlas
BioGRID	Biological interactions database across multiple species.	Multiple (yeast, fly, worm, human)	Maps interaction networks for understanding disease pathways.	BioGRID
Comparative Toxicogenomics Database (CTD)	Links chemicals, genes, and diseases across species.	Human, mouse, rat, zebrafish	Provides cross-species toxicogenomic data for understanding human diseases.	CTD
ModBase	Database of protein structure models for various species.	Multiple species	Structural insights into proteins related to human disease pathways.	ModBase
OrthoDB	Provides orthologous gene groups across species.	Multiple species	Identifies conserved genes and pathways relevant to human diseases.	OrthoDB
NCBI Datasets	Identifies homologous genes across eukaryotic species.	Multiple species	Enables comparison of gene sequences and functions; links cross-species gene-disease relationships.	NCBI Datasets
MONDO	Part of Monarch Initiative to harmonize disease definitions.	Disease ontology used alongside model research.	Comprehensive integration of disease entities.	Mondo

## Data Availability

No datasets were generated or analysed during the current study.
